# Effect of Brood Age on Nestling Diet and Prey Composition in a Hedgerow Specialist Bird, the Barred Warbler *Sylvia nisoria*

**DOI:** 10.1371/journal.pone.0131100

**Published:** 2015-06-26

**Authors:** Grzegorz Orłowski, Andrzej Wuczyński, Jerzy Karg

**Affiliations:** 1 Institute of Agricultural and Forest Environment, Polish Academy of Sciences, Poznań, Poland; 2 Institute of Nature Conservation, Polish Academy of Sciences, Lower-Silesian Field Station, Wrocław, Poland; 3 Department of Nature Conservation, Faculty of Biological Sciences, University of Zielona Góra, Zielona Góra, Poland; University of Hawaii at Manoa, UNITED STATES

## Abstract

The composition and quality of food provided to nestling birds influence their growth and development and offers key insight into the ecological requirements of birds. One bird species whose feeding ecology is poorly understood is the Barred Warbler (*Sylvia nisoria*), which utilizes semi-natural shrubby vegetation in agroecosystems. Because Barred Warbler nestlings vary greatly in body mass we hypothesised that diet and prey properties (size, diversity, taxonomic composition, and chitin content and resulting body hardness and digestibility) would differ as the nestlings aged. We quantified the diet based on faecal analysis, sampling faecal sacs from the nestlings pooled into three age classes: 2-3 days old, 4-6 d old, and 7-9 d old. Nestlings were provided a wide diversity of food and a strong relationship existed between food characteristics and nestling age. The youngest nestlings (2-3 d old) had the lowest values of each dietary characteristic (diversity, number and total biomass of prey, and individual prey weight), that were significantly lower than the oldest nestlings (7-9 d old). Nestlings aged 4-6 d exhibited intermediate dietary characteristics. Differences in dietary composition of the six major food types showed marked differences between the individual broods and age categories. Percentages of the number and biomass of soft-bodied prey were highest in the diet of 2-3 d and 4-6 d old nestlings, and decreased with increasing age, whereas the opposite trend was observed in the percentage of intermediately and heavily chitinised prey. Parent Barred Warblers probably preferentially select soft-bodied prey for the youngest nestlings, and satisfy the greater energy demands of the older ones by providing them with a greater variety of prey containing more chitin, as well as plant food. The provisioning of less-readily digestible prey to older nestlings suggests that as the quality of food decreases the quantity increases, implying that the youngest nestlings may be physiologically limited as regards their ability to digest more heavily chitinised prey.

## Introduction

Knowledge of the different food types provided to nestlings offers key insight into the ecological requirements of birds. During the ontogeny, the energy demands of growing nestlings increase considerably, and their growth requires changes in the amount and type of food provisioned by parents. However, these cost-benefit trade-offs in prey delivery to nestlings are dynamic and depend on both intrinsic and extrinsic factors (after [1]). Several studies have shown that the size of food items increases as nestlings mature. This suggests that the relative size of the food load can be adjusted to that of the young (reviewed in [2–5]). The age-related increase in prey size implies that the nutritional requirements of the nestlings are a primary determinant of adult foraging strategy [6]. Another potential explanation are the gape-size constraints, which principally limit the size of prey ingested [5–7]. Furthermore, growing nestlings show a progressive increase in their digestive efficiency [8], resulting from both the development of digestive organs and the production of digestive enzymes, which enable them to achieve the adult-like stage during the relatively short nestling period [9]. In insectivores, the development of digestive abilities reflects the growing tolerance to the chitin content of prey, affecting overall changes in diet characteristics, and possibly prey selection by parent birds [10–12].

Along with the physiological and/or morphological limitations, the dietary changes in nestlings are also directly affected by the availability and/or distribution of prey in the environment where the adult birds forage [11,13]. This dependence may be especially important in species inhabiting seasonally changing environments with fluctuating resources, such as agroecosystems [14]. Food resources in such habitats depend on landscape patchiness, especially the presence of non-crop vegetation, and also on farming activities, since the arthropods living in crops are the staple diet of farmland birds [15,16]. However, in European agricultural landscapes there are only a few true farmland species (i.e. open-field specialists); the majority of bird species found there are associated with the field-forest mosaic. Therefore, the presence of residual semi-natural habitats like field margins, hedgerows and tree lines are of critical importance for conserving the avian and overall biodiversity richness within agricultural landscapes [17–19]. More importantly, these habitats are suffering a dramatic decline at the continental scale as a result of the increase in intensive farming [20,21]. Unfortunately, the bulk of the ornithological literature relating to farmland birds documents population processes; dietary studies potentially giving valuable insight into the mechanisms of these processes and into the ecology of individual bird species are uncommon (reviewed in [22–23]). Such studies are especially necessary for poorly studied and/or declining species. There are few direct dietary studies of bird species associated with shrubby vegetation (such as various types of shelterbelts or hedgerows), and most of those were conducted in the geographically relatively limited area of north-western Europe [15–16,22–25].

In this paper we present, for the first time, the results of a dietary study of a species with a poorly known feeding and foraging ecology, the Barred Warbler *(Sylvia nisoria)*. The species breeds in spontaneous shrubby vegetation growing along linear habitat features such as unpaved roads and ditches. A network of these boundary structures (hereafter called field margins *sensu* [26]) is a typical feature of the extensive agricultural landscapes of central and eastern Europe (cf. [27–28]). Both the target habitat and the target species are of prime conservation concern in Europe. Field margins are the most common non-crop habitats in many farmlands and are thus the most important for farmland biodiversity conservation. The European breeding range of the Barred Warbler is limited to the central and eastern part of the continent: this constitutes >50% of its global breeding range [29]. Because of this large contribution and the loss and degradation of the species’ habitat, the Barred Warbler is listed in Annex 1 of The Birds Directive on the conservation of wild birds (Directive 2009/147/EC). This Sylviid warbler is accepted as a focal species of broad-leaved shrubby vegetation growing beyond typical forest habitats [27,30–37]. Therefore, taking previous habitat classifications of farmland birds into consideration (cf. [38–39]), we have categorised the Barred Warbler as a ‘hedgerow specialist’. Indeed, in low-intensity farming systems well-vegetated field margins interspersed with shrubs are the main breeding and foraging habitat of the Barred Warbler, and the species can breed in high densities in these residual habitats [27,36]. Despite the acknowledged importance of shrubby vegetation within the Barred Warbler’s breeding territories in farmland (cf. references above), no recent dietary data from these environments, supported by statistical treatment, exist for this warbler (reviewed in [32,40]). Accordingly, the main objective of the present study was to assess the food composition provisioned to nestling Barred Warblers and to examine the effect of brood age on diet. Since the nestlings varied greatly in body mass we hypothesised that diet and prey properties would differ as the nestlings aged. This hypothesis had previously been set up by some authors, but was based on insufficient data (cf. [32]). These differences may relate not only to the size, diversity and taxonomic composition of prey, but also to the variable contribution of different prey items according to their chitin content and resulting body hardness and digestibility. Finally, considering the relatively long range of foraging trips (up to 300–400 m from the nests) and the variety of foraging habitats used by parent Barred Warblers (such as various strata of shrubs, trees and soil surface, including cereal crops; [41–43], A. Wuczyński—unpubl.), we expected that the nestling diet might be differentiated taxonomically and contain a variety of animal taxa from these micro-habitats.

## Material and Methods

To address our main objective we studied Barred Warblers in a heterogeneous agricultural area of the Sudeten Foreland (altitude 200–250 m) in south-western Poland during four breeding seasons (2006–2008 and 2010). The area is characterised by a low-intensity arable farming system, covered by small, privately-owned fields (0.1 ha up to several hectares), and a network of linear, semi-natural field margins—the main breeding and foraging habitat of the Barred Warblers. Among the variety of field margins available (from herbaceous to tree-lined), the subset of shrubby margins was the species’ preferred habitat [27]. The shrubby margins occurred in a density of c. 6 km/km^2^, were usually located along a functional component (road, ditch), and comprised broad-leaved shrub species such as *Sambucus nigra*, *Prunus spinosa*, *Crataegus* ssp., *Rosa* ssp., some isolated, deciduous trees, such as *Fraxinus excelsior*, *Alnus glutinosa*, *Quercus robur*, or remnants of fruit tree lines, mostly *Prunus avium* or *Malus* sp. (more details on the vegetation structure, and the animal and plant communities inhabiting the field margins have been presented elsewhere: cf. [27–28], Wuczyński, submitted). The local density of the Barred Warbler in the study area was 0.54 pairs/10 ha at the landscape scale (six 50 ha study plots, 2004–2005, Wuczyński, submitted). At the habitat scale the ecological density in linear habitat features was 1.2 pairs/km of field margins with various vegetation types or 1.7 pairs/km of shrubby field margins [27].

The description of the diet of nestling Barred Warblers was based on faecal analysis. Faecal sacs were collected during an extensive study of the breeding biology of Barred Warblers (A. Wuczyński—unpubl.). The Barred Warbler nestlings from which the faecal sacs were sampled were aged according to parallel measurements of body weight and feather development of other individually marked nestlings in nests located in the same area. The nestlings were aged between 2 and 9 days, except for one brood in which the nestlings must have been older (10–12 days) when producing the faecal sacs (10 in total), as these were found below the nest after the nestlings had fledged. In most dietary analyses we pooled the nestlings into three age classes, corresponding to the differences in their body weight: 2–3 days old, nestling weight < 10 g (*n* = 17 faecal sacs); 4–6 d old, 10–15 g (*n* = 23); and 7–9 d old, above 15 g (*n* = 61). The last category also included the older nestlings from the brood mentioned above.

Overall, during four breeding seasons– 2006–2008 and 2010–101 faecal sacs were collected from 11 different broods. The number of faecal sacs/broods collected in consecutive years was: 2006 (33/3), 2007 (57/6), 2008 (7/1) and 2010 (4/1). Since some faecal sacs were collected from the same three broods on consecutive days, the dietary data for these broods were treated separately in various age categories in the further analysis. Overall, we sampled faecal sacs from four of the youngest broods (2–3 d), four 4–6 d old broods and six 7–9 d old broods. Our sampling dates ranged between 4 June and 17 July, including 49 faecal sacs sampled in the first half of June, 35 from the second half of June, and 17 from July. The average daily temperature (°C) and total precipitation (mm) for June-July was 19.9/99.4 (2006), 18.5/188.8 (2007), 18.0/94.0 (2008) and 18.6/144.7 (2010) (data obtained from the weather station in Sieniawka, 50°47’N and 16°47’E, within the study area).

### Diet analysis and further data treatment

Before analysis the droppings were crushed manually and separated on Petri dishes. The food components in the individual droppings were identified. Faecal analysis was performed using a binocular microscope at 40× magnification. The number of prey items representing particular invertebrate taxa was established from the numbers of fragments of chitin parts, chiefly the elytra (for different families and genera of Coleoptera, Homoptera or Heteroptera), wings (in the case of Diptera, Hymenoptera), mouthparts (most of the orders) and other preserved organs (e.g., limbs, petiolus, clypeus, mandible). During the determination of the number of prey items belonging to a particular taxon, a rule summing the different chitin parts to the level of one individual was applied: two or more different fragments of chitin parts (e.g., head, mandibles, six legs and other parts in the case of ants) from one dropping was treated as belonging to the same individual of a given species [43–44]. The mass of prey was calculated as dry mass (mg d.w.); these values were obtained from detailed measurements of insect weights based on the analysis of 479 087 individuals of different insect taxa [45], applied in an earlier dietary study on insectivorous passerines [43–44]. Since the diet of nestlings, especially the youngest ones, can differ from the older individuals with respect to the chitin content of the prey delivered, we arbitrarily categorised the identified prey taxa into three groups: soft-bodied prey (such as Arachnida, larvae), intermediately chitinised (Diptera, Plecoptera, Hymenoptera, Heteroptera and Orthoptera) and heavily chitinised prey (such as adult Coleoptera and small snail shells). The general dietary composition pertaining to nestlings in the three age classes and the total number of prey are presented in Table 1. Despite the bias in some results of the dietary composition in small insectivorous passerines, including Sylviids, based on faecal analysis, especially with respect to easy-digestible prey like caterpillars, Diptera and Hemiptera [46–47], the results of our study can be compared with each other within our sample (cf. [48]).

**Table 1:** The number of various prey taxa (with individual dry mass expressed in mg dry weight) identified in faecal sacs (*N* = 101) of Barred Warbler *Sylvia nisoria* nestlings in three age classes, 2–3 d old (*n* = 17 faecal sacs), 4–6 d old (*n* = 23) and 7–9 d old (*n* = 61) breeding in woody field margins in south-western Poland; ^1^chitin content: soft-bodied prey (s), intermediately chitinised (i), heavily chitinised (h).

Prey (chitin content)^1^			Nestling age (body mass)							
Class/order	Taxa/species	Dry mass (mg dw)	2–3 d old (<10 g)		4–6 d old (10–15 g)		7–9 d old (>15 g)		All nestlings	
			*n*	(%)	*n*	(%)	*n*	(%)	*n*	(%)
Arachnida	Araneae (s)	2.7	46	(59.0)	78	(52.0)	156	(32.6)	280	(39.6)
	Opiliones (s)	2.7	-	-	7	(4.7)	5	(1.0)	12	(1.7)
Coleoptera	Psylliodes (h)	1.8	-	-	-	-	41	(8.6)	41	(5.8)
	Agriotes (h)	9.7	-	-	5	(3.3)	24	(5.0)	29	(4.1)
	Coleoptera (h)	6.6	-	-	4	(2.7)	15	(3.1)	19	(2.7)
	Zabrus (h)	8.5	-	-	2	(1.3)	9	(1.9)	11	(1.6)
	Phyllobius (h)	3.7	-	-	2	(1.3)	7	(1.5)	9	(1.3)
	Curculionidae (h)	2.8	-	-	2	(1.3)	5	(1.0)	7	(1.0)
	Elateridae (h)	13.8	-	-	-	-	2	(0.4)	2	(0.3)
	Ophonus (h)	8.5	-	-	-	-	1	(0.2)	1	(0.1)
	Phyllopertha (h)	17.4	-	-	-	-	1	(0.2)	1	(0.1)
	Propylaea (h)	3.2	-	-	-	-	1	(0.2)	1	(0.1)
	Rhinchites (h)	4.7	-	-	1	(0.7)	-	-	1	(0.1)
	Bembidion (h)	1.2	-	-	-	-	1	(0.2)	1	(0.1)
	*Coccinella septempunctata* (h)	13.7	-	-	-	-	1	(0.2)	1	(0.1)
	Coleoptera larvae (s)	6.0	-	-	-	-	1	(0.2)	1	(0.1)
Diplopoda (h)		66.8		-	-	-	1	(0.2)	1	(0.1)
Diptera	Brachycera (s)	14.1	4	(5.1)	2	(1.3)	5	(1.0)	11	(1.6)
	unident. (i)	6.8	-	-	1	(0.7)	1	(0.2)	2	(0.3)
Heteroptera	Pentatomidae (i)	26.2	4	(5.1)	2	(1.3)	18	(3.8)	24	(3.4)
	*Palomena prasina* (i)	23.2	-	-	-	-	14	(2.9)	14	(2.0)
	Eurygaster (i)	36.3	-	-	-	-	7	(1.5)	7	(1.0)
	Miridae (i)	2.2	-	-	2	(1.3)	4	(0.8)	6	(0.8)
	Eurydema (i)	8.2	-	-	-	-	5	(1.0)	5	(0.7)
	Aelia (i)	14.3	-	-	-	-	4	(0.8)	4	(0.6)
	*Dolycoris baccarum* (i)	26.6	-	-	-	-	3	(0.6)	3	(0.4)
	Heteroptera (i)	9.7	-	-	1	(0.7)	1	(0.2)	2	(0.3)
	Dolycoris (i)	26.6	-	-	-	-	1	(0.2)	1	(0.1)
	Palomena (i)	42.0	-	-	-	-	1	(0.2)	1	(0.1)
Homoptera	Aphrophora (s)	6.5	-	-	-	-	1	(0.2)	1	(0.1)
Hymenoptera	Lasius (i)	0.6	1	(1.3)	6	(4.0)	11	(2.3)	18	(2.5)
	Ichneumonidae (i)	2.4	1	(1.3)	7	(4.7)	5	(1.0)	13	(1.8)
	Apidae (i)	19.8	-	-	3	(2.0)	6	(1.3)	9	(1.3)
	Formicidae (i)	0.6	-	-	-	-	2	(0.4)	2	(0.3)
	Formica (i)	1.2	-	-	1	(0.7)	-	-	1	(0.1)
	Myrmica (i)	1.2	-	-	-	-	1	(0.2)	1	(0.1)
	Pteromalidae (h)	0.2	1	(1.3)	-	-	-	-	1	(0.1)
	Vespula (h)	25.7	-	-	-	-	1	(0.2)	1	(0.1)
Lepidoptera	unident. larvae (s)	8.2	17	(21.8)	15	(10.0)	49	(10.2)	81	(11.5)
Plecoptera (s)		9.0	-	-	1	(0.7)	-	-	1	(0.1)
Orthoptera	Metrioptera (i)	134.6	-	-	-	-	14	(2.9)	14	(2.0)
	Tetrigidae (i)	11.4	2	(2.6)	-	-	5	(1.0)	7	(1.0)
	Tetrix (i)	11.4	-	-	-	-	2	(0.4)	2	(0.3)
Unidentified insects		-	2	(2.6)	7	(4.7)	22	(4.6)	31	(4.4)
Mollusca (h)		150.0	-	-	1	(0.7)	10	(2.1)	11	(1.6)
Plant seeds							13	(2.7)	13	(1.8)
	*Padus avium*	-	-	-	-	-	11	(2.3)	11	(1.6)
	*Rubus* sp.	-	-	-	-	-	2	(0.4)	2	(0.3)
Other plant remains (fruits)		-	-	-	-	-	2	(0.4)	2	(0.3)
TOTALS		-	78	(100)	150	(100)	479	(100)	707	(100)

The main dietary analysis was conducted for prey items identified in individual faecal sacs. For each faecal sac we determined three main dietary characteristics (diet diversity expressed as the number of prey taxa; total number of prey and total biomass of prey) and the composition of the diet expressed as the number and biomass of six major food types consumed (class/order of various invertebrates) and three groups of prey with different chitin content. Individual prey weight was calculated for the entire set of prey items without consideration of the ‘individual faecal sac’. In order to perform the statistical analysis six major food types were selected, representing taxonomically and morphologically related taxa: Arachnida, Coleoptera, Hemiptera (= Heteroptera + Homoptera), lepidopteran larvae (caterpillars), Diptera/Hymenoptera (including one single specimen of Plecoptera) and other invertebrates (Orthoptera, Mollusca, Diplopoda and unidentified insects). The dietary composition of the six major food types, as well as the three groups of prey categorised according to the chitin content, were presented as four dietary variables: number of prey; percentage of prey; biomass of prey; and percentage of prey biomass.

The key objective of our statistical analyses was to assess differences in the main dietary characteristics, including diet division into six major food types and three groups of prey with differing chitin content, between nestlings in the three age classes. Initially, the differences in the main dietary characteristics (after logarithmic or square root-arcsine*-*transformed percentage data also applied in multivariate analysis of variance, MANOVA) between nestlings in the three age classes were tested using ANOVA/MANOVA with nested design: the independent variables were the age of nestlings and the individual brood identity (nested within age of nestlings). The *post-hoc* contrast for assessing the differences for one individual prey group applied Tukey’s test with the Spjøtvoll-Stoline modification for unequal sample sizes [49]; then the same test procedure was applied in MANOVA. Non-parametric ANOVA Kruskal-Wallis [50] tests were applied to assess the differences in individual prey weight calculated for the entire set of prey items without consideration of the ‘individual faecal sac’ approach.

MANOVA was applied to assess differences in dietary composition (expressed as the six major food types and the three groups of prey with differing chitin content) between nestlings in three age classes, considering also the brood identity nested within the age of nestlings [50]. It is noteworthy that in dietary studies of predatory species, including insectivorous birds, the biomass approach is considered to be of greater relevance for characterising the type of prey consumed than a mere numerical quantification of the prey [2,51–52]. Statistical analyses were conducted with the aid of Statistica 7.0 [50] and Excel software. The probability of *P* < 0.05 was considered to be statistically significant.

### Ethics Statement

The field study was conducted on a total area of c. 400 km^2^ (see [28]). The coordinates of the rough mid-point of the area were 50°47’N and 16°47’E. In this area the nests of Barred Warblers were found in hedgerows located along roads and ditches which are open to the public, so no special permits were required for access to these locations. No protected species were sampled during the field study; only the faeces of the protected species, the Barred Warbler, were collected. We did not need to apply to the animal ethics committee for any special approval for these activities because the faecal sacs of Barred Warblers were collected during standard bird ringing procedures. The rules of bird ringing in Poland are determined by superior regulations, i.e. the nature conservation act (Ustawa z dnia 16 kwietnia 2004 r. o ochronie przyrody, (Dz. U. z 2013 r. poz. 627, z późn. zm.), the regulation on bird ringing (Rozporządzenie Ministra Środowiska z dnia 14 marca 2006 r. w sprawie obrączkowania ptaków (Dz. U. Nr 48, poz. 350), and the rules of the Polish Bird Ringing Centre. The last-mentioned rules (www.stornit.gda.pl/reg_kco.php) set out the details of the standard ringing procedures. In accordance with section III, point 1 of these rules, licensed ringers are permitted to trap and ring wild birds. During ringing procedures, passerine nestlings frequently excrete faeces, which can be collected by the ringer. Since all nestling Barred Warblers were ringed in accordance with the above regulations and no approval is necessary to collect faeces of wild birds, we did not seek to obtain any special approval from the animal ethics committee in this respect.

## Results

Overall, we identified 692 different animal prey items in all the faecal sacs, including 661 prey with a known individual mass (Table 1). The mean (95% CI) weight calculated for the entire set of prey items was 11.8 (9.7–13.8) mg dw. The most numerous prey group was Arachnida (spiders and harvestmen)– 41.3% by number in total (Table 1). However, the overall numerical contribution of Arachnida varied with the age of nestlings and was relatively highest in the youngest ones (age 2–3 d) (Table 1).

### Diet of nestlings in relation to their age

**Fig 1:**
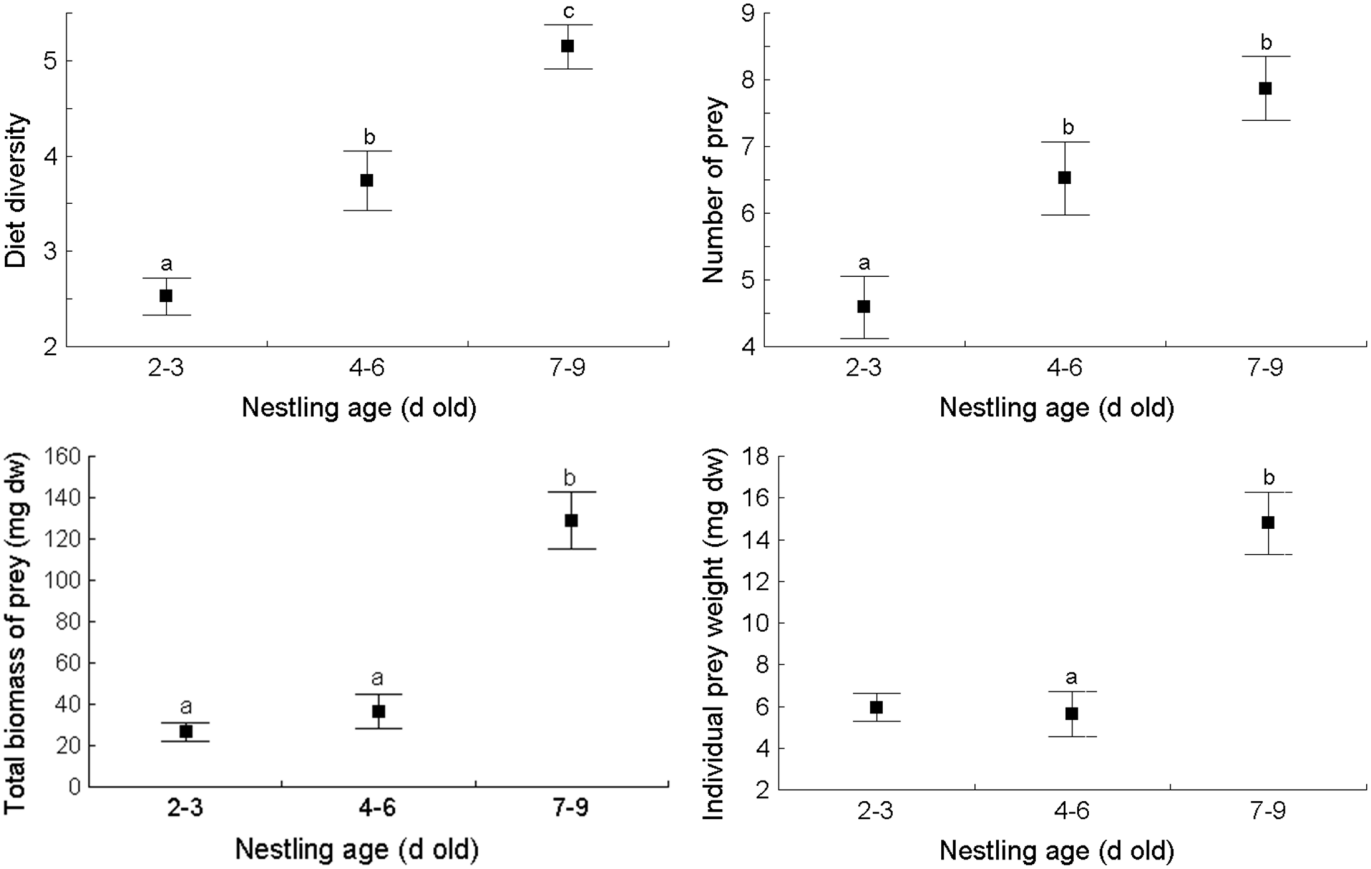
The main dietary characteristics (average ± SE) of nestling Barred Warblers *Sylvia nisoria* in three age classes. The various letters indicate statistically significant differences obtained in the *post-hoc* comparison with ANOVA or ANOVA Kruskal-Wallis (see Results section for details).

The four main dietary characteristics calculated for individual faecal sacs showed statistically significant differences associated with brood identity and nestling age: diet diversity expressed as the number of prey taxa, the number of prey items, the total biomass of prey (Table 2) and individual prey weight (calculated for the entire set of prey items) (ANOVA Kruskal-Wallis, H_2,661_ = 13.0, P = 0.015) (Fig 1). In most cases the youngest, 2–3 d old nestlings showed the lowest values of each dietary characteristic, which were significantly lower compared to 7–9 d old nestlings. The most pronounced differences were found for the total biomass of prey, which was nearly 6-fold higher in the oldest nestlings than in the youngest ones. Further, dietary diversity and number of prey in faecal sacs of 2–3 d old nestlings were approximately two-fold lower compared to the oldest ones. Nestlings aged 4–6 d displayed a relatively intermediate dietary characteristics. Exact patterns of between-group differences are presented in Fig 1.

**Table 2:** Results of ANOVA (type III ss) testing the effect of age and brood identity (nested within age) of nestlings on main dietary characteristics in Barred Warbler *Sylvia nisoria* nestlings.

Source of variation	Age			Brood identity (Age)		
	df	F	*P*	df	F	*P*
Diet diversity	2	24.69	<0.0001	11	4.60	<0.0001
Number of prey items	2	10.13	<0.0001	11	8.86	<0.0001
Total biomass of prey	2	17.62	<0.0001	11	4.22	<0.0001

**Fig 2:**
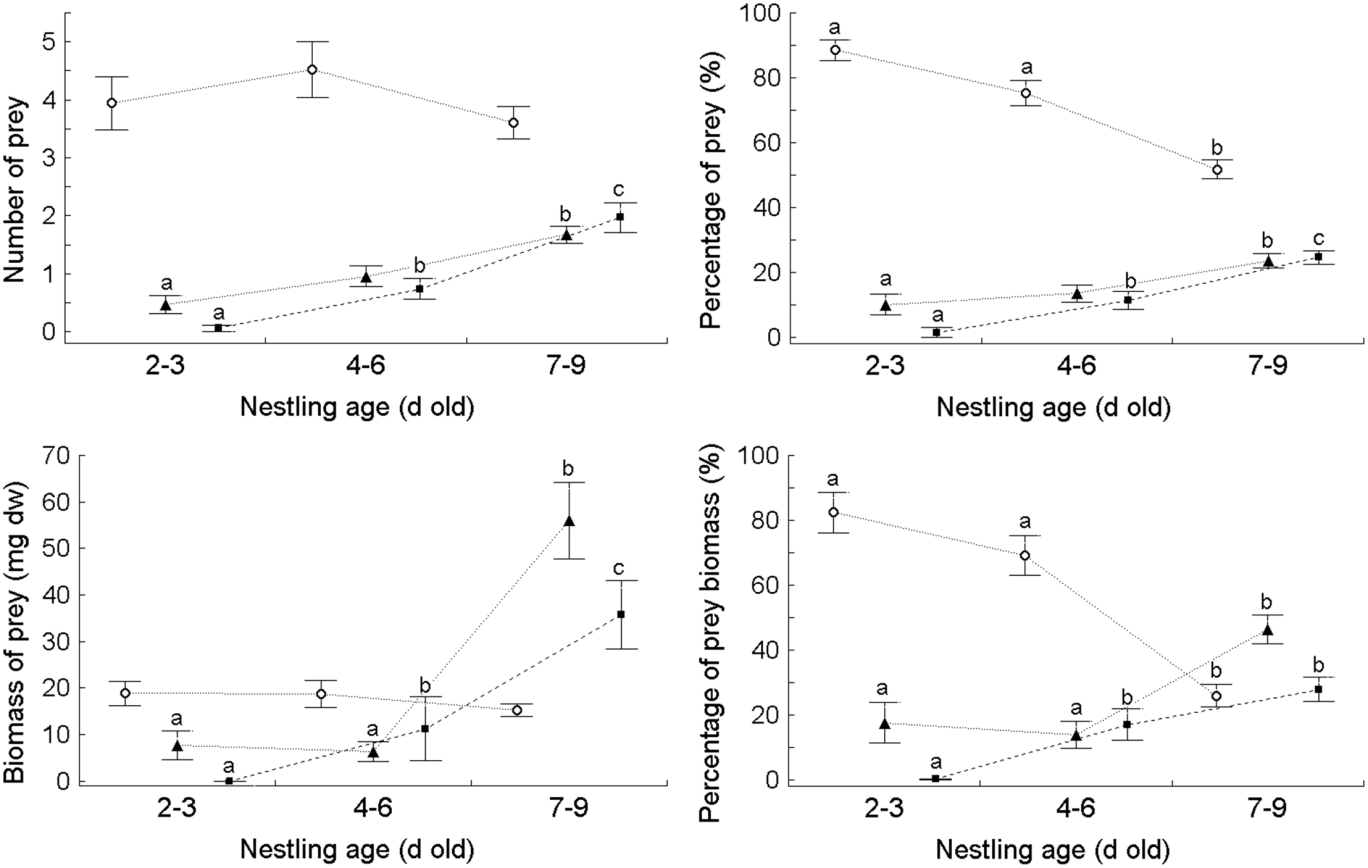
The average (±SE) number and biomass of three types of prey in relation to chitin content, soft-bodied prey (○), intermediately chitinised (▲) and heavily chitinised (■), identified in individual faecal sacs (*N* = 101) in three age classes of nestling Barred Warblers *Sylvia nisoria*. The various letters indicate statistically significant differences obtained in the *post-hoc* comparison with MANOVA between (see Table 4); the lines connect the same prey groups.

MANOVA applied to assess the overall differences in dietary composition expressed as four dietary variables (each representing the six major food types) showed marked differences between the three age categories of Barred Warbler nestlings and between individual broods (nested within the age category) for each variable: number of prey, percentage of prey, total biomass of prey and percentage of prey biomass (Table 3). Similarly, MANOVA showed significant differences in the four dietary variables expressing the chitin content (Fig 2, Table 4).

**Table 3:** Results of MANOVA testing the effect of age and brood identity (nested within age) of nestlings on dietary composition expressed as four dietary variables (each representing the six major food types: Arachnida, Coleoptera, Hemiptera, Lepidoptera larvae, Diptera/Hymenoptera and other invertebrates) in Barred Warbler *Sylvia nisoria* nestlings.

Source of variation	Age					Brood identity (Age)				
	Wilk’sλ	df		F	*P*	Wilk’sλ	df		F	*P*
		H_o_	Error				H_o_	Error		
Number of prey	0.364	12	164.0	8.97	<0.0001	0.115	66	444.2	3.35	<0.0001
Percentage of prey	0.347	12	164.0	9.53	<0.0001	0.163	66	444.2	2.72	<0.0001
Total biomass of prey	0.357	12	164.0	9.21	<0.0001	0.121	66	444.2	3.25	<0.0001
Percentage of prey biomass	0.301	12	164.0	11.23	<0.0001	0.151	66	444.2	2.85	<0.0001

**Table 4:** Results of MANOVA testing the effect of age and brood identity (nested within age) of nestlings on dietary composition expressing the chitin content in prey of Barred Warbler *Sylvia nisoria* nestlings (see Fig 2).

Source of variation	Age					Brood identity (Age)				
	Wilk’sλ	df		F	*P*	Wilk’sλ	df		F	*P*
		H_o_	Error				H_o_	Error		
Number of prey	0.552	6	170.0	9.80	<0.0001	0.323	33	251.1	3.56	<0.0001
Percentage of prey	0.496	6	170.0	11.88	<0.0001	0.324	33	251.1	3.55	<0.0001
Total biomass of prey	0.480	6	170.0	12.58	<0.0001	0.383	33	251.1	2.93	<0.0001
Percentage of prey biomass	0.433	6	170.0	14.72	<0.0001	0.315	33	251.1	3.66	<0.0001

Furthermore, MANOVA was applied to assess the comparisons for the four dietary variables expressing the chitin content (Fig 2) considering only two age classes of nestlings (not depicted), i.e. 2–6 d old (= pooled 2–3 d old and 4–6 d old nestlings) and 7–9 d old nestlings, still showed highly significant differences between these two groups of nestlings (MANOVA, in each case *P* ≤ 0.0004; for Age, df = 3 and 87.0; and for Brood identity(Age), df = 3 and 256.04).

Overall, *post-hoc* comparisons for the individual dietary variables showed that younger nestlings (2–3 d old and 4–6 d old), received significantly fewer (expressed as real numbers consumed) heavily chitinised prey items, primarily Coleoptera and Hemiptera, than the older 7–9 d old nestlings (Table 5 and Fig 2). No Coleoptera were found in the diet of 2–3 d old nestlings; the numbers and biomass of Coleoptera in the diet clearly increased with the age of nestlings, as was the case with Hemiptera (Table 1 and Table 5). On the other hand, the percentages of numbers and biomass of soft-bodied prey consumed, such as Arachnida and lepidopteran larvae, were the highest in the diet of 2–3 d old and 4–6 d old nestlings (Table 5), and clearly decreased with increasing nestling age, resulting in significant differences between the oldest nestlings and the other two age categories (Fig 2). A contrary trend was observed in the case of the percentage of intermediately and heavily chitinised prey, the contribution of which increased with the nestling age; there were marked differences between consecutive age classes especially in the case of the latter group (Fig 2).

**Table 5:** The average (±SE) number and biomass of four dietary variables expressing the six major food types (class/orders of invertebrates) identified in individual faecal sacs (*N* = 101) of nestling Barred Warblers *Sylvia nisoria* in three age classes, 2–3 d old (*n* = 17 faecal sacs), 4–6 d old (*n* = 23) and 7–9 d old (*n* = 61); the various letters indicate statistically significant differences between various age classes obtained in the *post-hoc* comparison with MANOVA (see Table 3).

Dietary variable/food type	Nestling age			
	2–3 d old	4–6 d old	7–9 d old	All nestlings
NUMBER OF PREY				
Arachnida	2.7 (±0.3)	3.5 (±0.4)	2.6 (±0.2)	2.9 (±0.2)
Coleoptera	0.0 (±0.0)^A^	0.7 (±0.2)^B^	1.8 (±0.3)^C^	1.2 (±0.2)
Hemiptera	0.2 (±0.1)^A^	0.2 (±0.1)^A^	1.0 (±0.1)^B^	0.7 (±0.1)
Lepidoptera larvae	1.0 (±0.2)	0.7 (±0.1)	0.8 (±0.1)	0.8 (±0.1)
Diptera/Hymenoptera	0.4 (±0.1)^A^	1.1 (±0.3)^B^	0.5 (±0.1)	0.6 (±0.1)
Other invertebrates	0.2 (±0.1)^A^	0.3 (±0.1)^A^	1.1 (±0.1)^B^	0.8 (±0.1)
PERCENTAGE OF PREY				
%Arachnida	60.1 (±4.2)^A^	55.4 (±4.3)^A^	34.0 (±2.2)^B^	43.3 (±2.1)
%Coleoptera	0.0 (±0.0)^A^	9.9 (±2.4)^B^	20.4 (±2.0)^C^	14.6 (±1.5)
%Hemiptera	4.4 (±2.0)^A^	3.0 (±1.2)^A^	13.6 (±1.6)^B^	9.7 (±1.2)
%Lepidoptera larvae	22.2 (±4.3)^A^	9.6 (±2.3)^B^	11.2 (±1.7)	12.7 (±1.4)
%Diptera/Hymenoptera	8.7 (±3.1)	13.8 (±2.7)^A^	6.3 (±1.2)^B^	8.4 (±1.1)
%Other invertebrates	4.5 (±2.0)^A^	8.3 (±3.1)	14.5 (±1.7)^B^	11.4 (±1.3)
BIOMASS OF PREY (mg d.w)				
Arachnida	7.4 (±0.8)	9.7 (±1.1)	7.2 (±0.6)	7.8 (±0.5)
Coleoptera	0.0 (±0.0)^A^	4.8 (±1.3)^B^	9.8 (±1.1)^C^	7.0 (±0.8)
Hemiptera	6.2 (±2.8)^A^	2.9 (±1.6)^A^	21.7(±2.1)^B^	14.8 (±1.6)
Lepidoptera larvae	8.2 (±2.0)	5.3 (±1.2)	6.6 (±1.0)	6.5 (±0.8)
Diptera/Hymenoptera	3.5 (±1.9)	7.3 (±2.3)	4.0 (±1.1)	4.7 (±0.9)
Other invertebrates	1.3 (±0.9)^A^	6.5 (±6.5)^A^	79.6 (±12.5)^B^	49.8 (±8.5)
PERCENTAGE OF PREY BIOMASS				
%Arachnida	37.4 (±5.7)^A^	45.3 (±6.5)^A^	12.9 (±2.7)^B^	24.4 (±2.8)
%Coleoptera	0.0 (±0.0)^A^	13.8 (±4.0)^B^	12.9 (±2.0)^B^	10.9 (±1.6)
%Hemiptera	12.2 (±5.6)^A^	4.1 (±2.0)^A^	25.0 (±3.5)^B^	18.1(±2.5)
%Lepidoptera larvae	36.1 (±7.3)^A^	16.5 (±4.0)^B^	10.5 (±2.0)^B^	16.2 (±2.1)
%Diptera/Hymenoptera	10.7 (±4.8)	17.1 (±4.1)^A^	3.6 (±1.0)^B^	7.9 (±1.5)
%Other invertebrates	3.7 (±2.6)^A^	3.2 (±3.2)^A^	35.2 (±4.7)^B^	22.6 (±3.4)

## Discussion

The presented results confirmed our hypothesis that the properties of diet and prey in nestling Barred Warblers change as the young birds grow. In particular, these differences were related both to the increase in size and taxonomic diversity of prey, including the proportion of more heavily chitinised prey. Our study showed that the pronounced differences in both the four dietary characteristics (diversity, number, biomass and weight of individual prey) and the dietary composition (expressed as the number and biomass of six major food types and prey hardness) in Barred Warbler nestlings are related to their different ages; most likely they are a consequence of significant differences in body size and the increasing ability to digest more heavily chitinised prey. The findings are a good illustration of a basic aspect of early nestling diet in the development of small terrestrial insectivorous passerines, especially in the context of the key role of small and soft-bodied prey [53] (reviewed in [3–5]). These prey are relatively easily digestible, and spiders are rich in taurine, an amino acid essential for the proper development of the visual system and the cognitive function of neonates in both birds and mammals [5,54–55].

However, our results also suggest more complex relationships between nestling age and the composition of prey varying in digestibility (cf. Table 5 and Fig 2). The proportion of soft-bodied prey in the three age classes of nestlings was generally less diversified than that of “hard” prey; significant differences were noted only in percentage terms and not in absolute values of numbers and biomass of soft-bodied prey. In contrast, the amount of heavily chitinised prey increased in both absolute and percentage numbers. Moreover, the dietary properties of the intermediate age class were more similar to the youngest than the oldest nestlings. Several conclusions can be drawn from these results.

Firstly, soft-bodied prey are an important component of the diet throughout the nestling period in the Barred Warbler. Although such prey makes up the majority of the food delivered to the youngest nestlings, and the percentage of soft-bodied prey decreases as they grow, it still makes a sizeable contribution (arachnids– 34% and caterpillars– 11% in the oldest age class). It is unclear whether the minute amount of “hard” prey delivered to the youngest nestlings (mostly some Hemiptera) should be treated as an indication of physiological limitations to their abilities to digest more heavily chitinised prey, or whether this might point to some nutritional properties of soft-bodied prey that are essential during early development. Marked differences in the overall contribution of soft-bodied prey types between nestlings and adult passerines have been described in several species (cf. [12]).

Secondly, the greater variability in the food composition of the oldest nestlings, with large amounts of heavily chitinised prey, illustrates a trade-off between the quantity and quality of food delivered to the nest and a flexibility in parental foraging strategies [56]. Parent Barred Warblers are able to adjust their food allocation strategy to satisfy the greater energy demands of large chicks at the expense of nutritional quality, i.e. a greater proportion of less-easily digestible invertebrates as well as plant food.

Thirdly, there was a weak difference in the dietary indices between the first two age classes (2–3 and 4–6 d old), and a notable difference between these classes and the oldest nestlings. Moreover, we observed that only 2–3 d old nestlings from various broods received similar amounts of the three prey groups differing in chitin content. This suggests that somewhere in the middle of the nestling period there is a dietary threshold, i.e. a development stage when less-easily digestible food items can start to be delivered. Instead of our nominal data (pertaining to only three age classes), more regular measurements of changes in food composition as the young birds grow are necessary to establish that precise threshold (if it does indeed exist). The results of our study suggest that already +7 d old nestlings (which have a relatively large spectrum of food items; Table 1) have a similar diet to that of adult Barred Warblers, especially in the context of the contribution of heavily chitinised prey (Coleoptera 20%, Hemiptera 14%–mean per individual faecal sac; Table 5). Similarly, in Blue Tits *Cyanistes caeruleus* the percentage of large caterpillars at the age of seven days was comparable to that observed in subsequent days of the nestling period [5]. Therefore, it has been proposed that the gape-size constraint hypothesis relates principally to immediately post-hatch nestlings (1–4 d old), whereas nestlings older than 7 d are not limited by gape size (discussed in [5]). Our data on the Barred Warbler also suggest that the gape-size limitation is applicable only to the youngest nestlings.

We believe that the values obtained in our study, which are absolute (not percentage) values, like the number and biomass of prey, can be treated as a proxy for the body mass / gape size of nestlings or the volume of ingested food (≈ digestive capacity). On the other hand, the percentage values (both the number and biomass of prey) can presumably be treated as an indirect measure of food selectivity by parent Barred Warblers or their territory quality. The percentage values of prey consumed are also an important way of expressing the differences in the nutritional composition of various food items, as the ratio of protein to carbohydrate content can decrease with a brood’s growing energy demands [56]. More importantly, the revealed strong relationship between the age of nestlings and the number, biomass and individual mass of prey implies that to obtain a full picture of diet in young birds, one needs to have a cross-section of all age categories of young. Finally, it should acknowledged that since we used contractual and dry weights (which might be up to 80% lower than wet weights) to calculate the weight of soft-bodied Arachnida and lepidopteran larvae, it is very likely that none of the biomass values presented here represent the actual mass of prey consumed, which in practice means that the consumed biomass of soft-bodied prey was considerably higher, especially in younger nestlings. This makes some interpretations difficult, for example, testing the gape-size constraint hypothesis (see above).

Our findings largely confirmed our initial prediction of a large diversity of food in Barred Warbler nestlings varying in age. This diversity was also related to the differences between various broods; actually the models pertaining to most dietary indices revealed significant effects of brood identity on prey diversity (Tables 2–4). The result probably reflects, first, a wide heterogeneity of the agricultural landscapes in which the study was conducted. A mosaic land-use pattern with a dense network of various field margins and other non-cropped habitats allows the Barred Warblers to breed in high densities, but the breeding territories are likely to be different regarding the food supply and availability. Second, the variety of food observed in Barred Warbler nestlings can be explained by the variety of foraging modes, including aerial- and ground-feeding, undertaking long trips to distant foraging grounds, and covering a diversity of foraging habitats, especially the various patches of shrubby vegetation (A. Wuczyński—unpubl.). For instance, we observed a relatively large contribution of typically ground-dwelling insects, such as ants and some beetles (cf. Table 1), which rarely occur in the diet of nestlings of species foraging in shrubby vegetation. For example, no ants were recorded in a careful study of the related Whitethroat *Sylvia communis* [24,47]. In turn, such prey can be numerous in typical ground-foragers like Stonechats *Saxicola torquata* [48,57] or Black Redstarts *Phoenicurus ochruros* [58]. Additionally, the presence of various flying insects, like flies or hymenopterans, indicates the ability of Barred Warblers to catch insects on the wing [32] or alternatively, to catch them when they are less active at low temperatures [59]. Moreover, it seems that some large prey types, such as the large-bodied Orthopterans reported in the diet of oldest Barred Warbler nestlings (cf. Table 1), could have been provisioned to them after prior preparation (= removal of wings and legs). Interestingly, our study confirmed that nestling Barred Warblers were fed fruits of some fleshy-fruited shrubs such as Bird Cherry or brambles. Plant food components delivered to the nestlings were previously observed in this and related Sylviid warblers breeding in Europe, yet such data are highly deficient [32,60].

Summarising our findings and earlier literature data on the habitat requirements of Barred Warblers breeding in open farmland, the species could be classified as a multi-patch shrubby habitat user [31,34,36]. It accepts both sparse bushes and large patches of shrubs covering the field margin network, and it can also forage in open farmland (crops). However, because of its rather secretive behaviour and mainly eastern European distribution, the Barred Warbler remains one of the less well known warblers in Europe [61]. This also applies to the species’ foraging ecology. We therefore advocate further dietary studies to evaluate the effect of habitat configuration (such as the coverage of crops and shrubby vegetation) on the various dietary indices and prey properties, and its consequences for the growth of nestlings of Barred Warblers and other birds living in patches of semi-natural vegetation. Such studies are especially important in regions of low-intensity agricultural systems with a retained heterogeneous landscape structure supporting large populations of this and other hedgerow specialist birds. The richness of semi-natural habitats in such regions includes some declining invertebrate groups (such as spiders, harvestmen or some beetles), which are the staple diet of hedgerow specialist birds. The results of studies from such regions make a valuable contribution to comparisons with areas of intensive farming, where the gradual disappearance of these prey groups associated with the tendency towards agricultural intensification ([62–64] and references therein) may directly affect bird species. However, these processes need a better understanding supported by more detailed data on diet and foraging ecology.
